# Simultaneous Determination of Eight Ginsenosides in Rat Plasma by Liquid Chromatography–Electrospray Ionization Tandem Mass Spectrometry: Application to Their Pharmacokinetics

**DOI:** 10.3390/molecules201219790

**Published:** 2015-12-03

**Authors:** Li-Yuan Ma, You-Bo Zhang, Qi-Le Zhou, Yan-Fang Yang, Xiu-Wei Yang

**Affiliations:** State Key Laboratory of Natural and Biomimetic Drugs, Department of Natural Medicines, School of Pharmaceutical Sciences, Peking University Health Science Center, Peking University, No. 38, Xueyuan Road, Haidian District, Beijing 100191, China; maliyuan0506@126.com (L.-Y.M.); zybo5288@163.com (Y.-B.Z.); zqlzdn@163.com (Q.-L.Z.); yangyanfang@bjmu.edu.cn (Y.-F.Y.)

**Keywords:** stems-leaves of *Panax ginseng*, ginsenoside, pharmacokinetics, LC-ESI-MS/MS

## Abstract

A high-performance liquid chromatography–electrospray ionization tandem mass spectrometry (LC-ESI-MS/MS) method was successfully developed and validated for the identification and determination of eight ginsenosides: ginsenoside Rg_1_ (**1**); 20(*S*)-ginsenoside Rh_1_ (**2**); 20(*S*)-ginsenoside Rg_2_ (**3**); 20(*R*)-ginsenoside Rh_1_ (**4**); 20(*R*)-ginsenoside Rg_2_ (**5**); ginsenoside Rd (**6**); 20(*S*)-ginsenoside Rg_3_ (**7**); and 20(*R*)-ginsenoside Rg_3_ (**8**) in rat plasma. The established rapid method had high linearity, selectivity, sensitivity, accuracy, and precision. The method has been used successfully to study the pharmacokinetics of abovementioned eight ginsenosides for the first time. After an oral administration of total saponins in the stems-leaves of *Panax ginseng* C. A. Meyer (GTSSL) at a dose of 400 mg/kg, the ginsenosides **6**, **7**, and **8**, belonging to protopanaxadiol-type saponins, exhibited relatively long *t*_max_ values, suggesting that they were slowly absorbed, while the ginsenosides **1**–**5**, belonging to protopanaxatriol-type saponins, had different *t*_max_ values, which should be due to their differences in the substituted groups. Compounds **2** and **4**, **3** and **5**, **7** and **8** were three pairs of *R*/*S* epimerics at C-20, which was interesting that the *t*_1/2_ of 20(*S*)-epimers were always longer than those of 20(*R*)-epimers. This pharmacokinetic identification of multiple ginsenosides of GTSSL in rat plasma provides a significant basis for better understanding the clinical application of GTSSL.

## 1. Introduction

**Figure 1:**
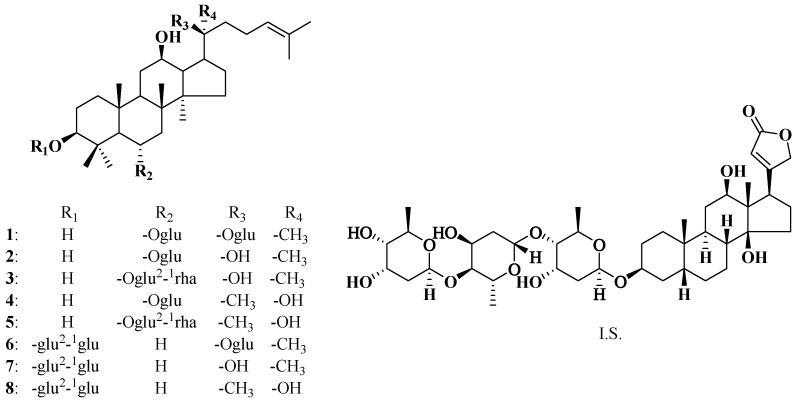
Chemical structures of eight ginsenosides (**1**, ginsenoside Rg_1_; **2**, 20(*S*)-ginsenoside Rh_1_; **3**, 20(*S*)-ginsenoside Rg_2_; **4**, 20(*R*)-ginsenoside Rh_1_; **5**, 20(*R*)-ginsenoside Rg_2_; **6**, ginsenoside Rd; **7**, 20(*S*)-ginsenoside Rg_3_; **8**, 20(*R*)-ginsenoside Rg_3_) and I.S. (digoxin).

*Panax ginseng* C. A. Meyer has been used as a medicinal plant in China for thousands of years to enhance stamina and capacity to cope with fatigue and physical stress. Current worldwide use has been diverse because of the molecular diversity in the chemical ingredients of *P. ginseng*, with mainly focused study on prevention and treatment of cardiovascular and cerebrovascular diseases. There are active chemical components called ginseng saponins (ginsenosides) in *P. ginseng*, which has been reported to be responsible for the ginseng’s medicinal properties [1]. A lot of studies on ginsenosides have mainly focused on the roots and rhizomes of *P. ginseng* because the aerial parts, including the stems and leaves, are usually discarded. In recent years, we conducted exploratory research on the chemical ingredients in the stems-leaves of *P. ginseng* harvested annually, and demonstrated that composition of total saponins in the stems and leaves of *P. ginseng* (GTSSL) [2,3,4,5] is not different from those of the roots and rhizomes of *P. ginseng* [6,7]. GTSSL contained ginsenoside in the chemical structure, such as ginsenosides Rh_1_ and Rg_3_, and is currently investigated as an anti-aging agent at the preclinical research stage. During the last decade, absorption, distribution, metabolism, excretion, and toxicity (ADMET) have been introduced into the earlier stages of drug discoveries instead of a serial of strategy, because ADMET and pharmacokinetic (PK) issues are partly responsible for failure in clinical trials. Although PK characteristics of individual 20(*R*)- and 20(*S*)-ginsenosides Rg_2_, which are the main ingredients in the GTSSL extract, has been reported after the intravenous dose to rats [8], there has been no any literature reporting PK behaviors of multiple ginsenosides in the GTSSL extract. It is therefore important to investigate PK characteristics of the main active components in GTSSL. The main purpose of this subject is to establish and validate a rapid, accurate, precise, sensitive, and selective high-performance liquid chromatography/electrospray ionization tandem mass spectrometer (LC-ESI-MS/MS) for the identification and quantification of the main active ginsenosides of GTSSL in rat plasma including ginsenoside Rg_1_ (**1**) [9,10,11,12], 20(*S*)-ginsenoside Rh_1_ (**2**) [13,14,15,16,17], 20(*S*)-ginsenoside Rg_2_ (**3**) [12,18,19,20], 20(*R*)-ginsenoside Rh_1_ (**4**), 20(*R*)-ginsenoside Rg_2_ (**5**) [18], ginsenoside Rd (**6**) [21,22,23], 20(*S*)-ginsenoside Rg_3_ (**7**) [12,24,25,26], and 20(*R*)-ginsenoside Rg_3_ (**8**) [27,28]. Their chemical structures are shown in Figure 1. The PK profiles of the eight ginsenosides could be revealed after an oral administration of GTSSL at a single dose of 400 mg/kg to rats based on the developed method. To our knowledge, it is the first study on the detailed PK characterizations of the less-polar eight main ginsenosides with diverse biological and pharmacological activities in GTSSL in rat plasma. It has been well known that the efficacy of ginsenoside increases with the extent of less-polar molecules or deglycosylation, which enhances its hydrophobicity and ability to permeate the cell wall.

## 2. Results and Discussion

### 2.1. Identification and Determination of Ginsenosides in Rat Plasma

**Figure 2:**
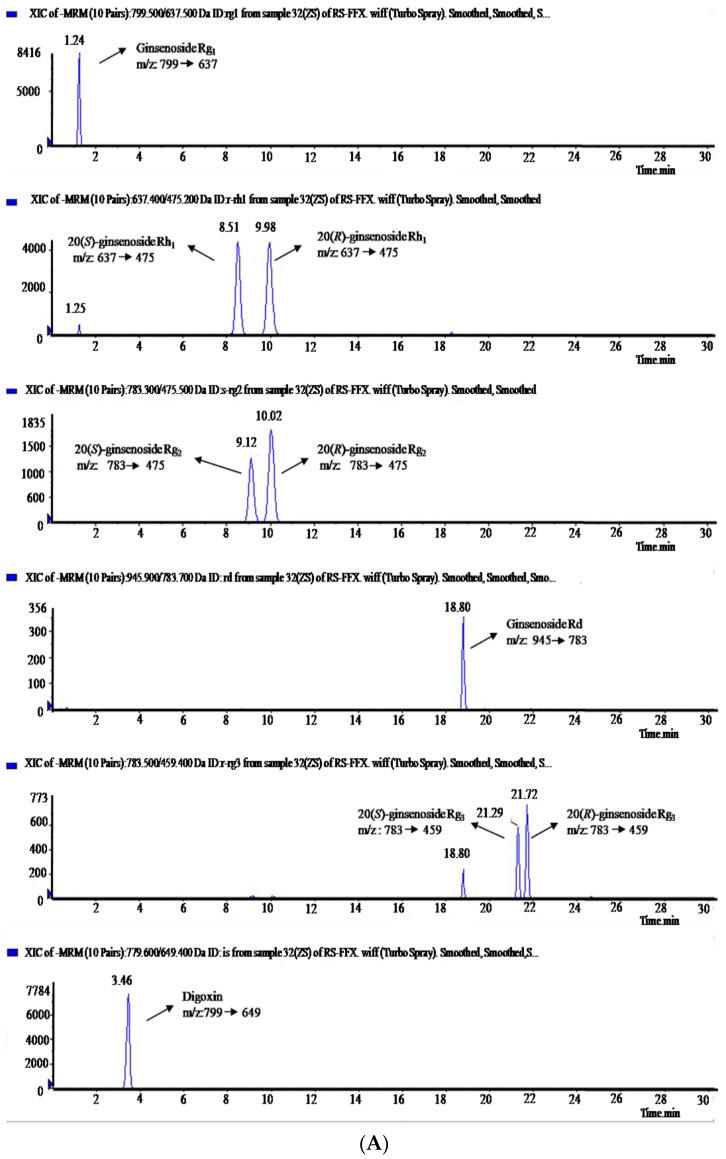

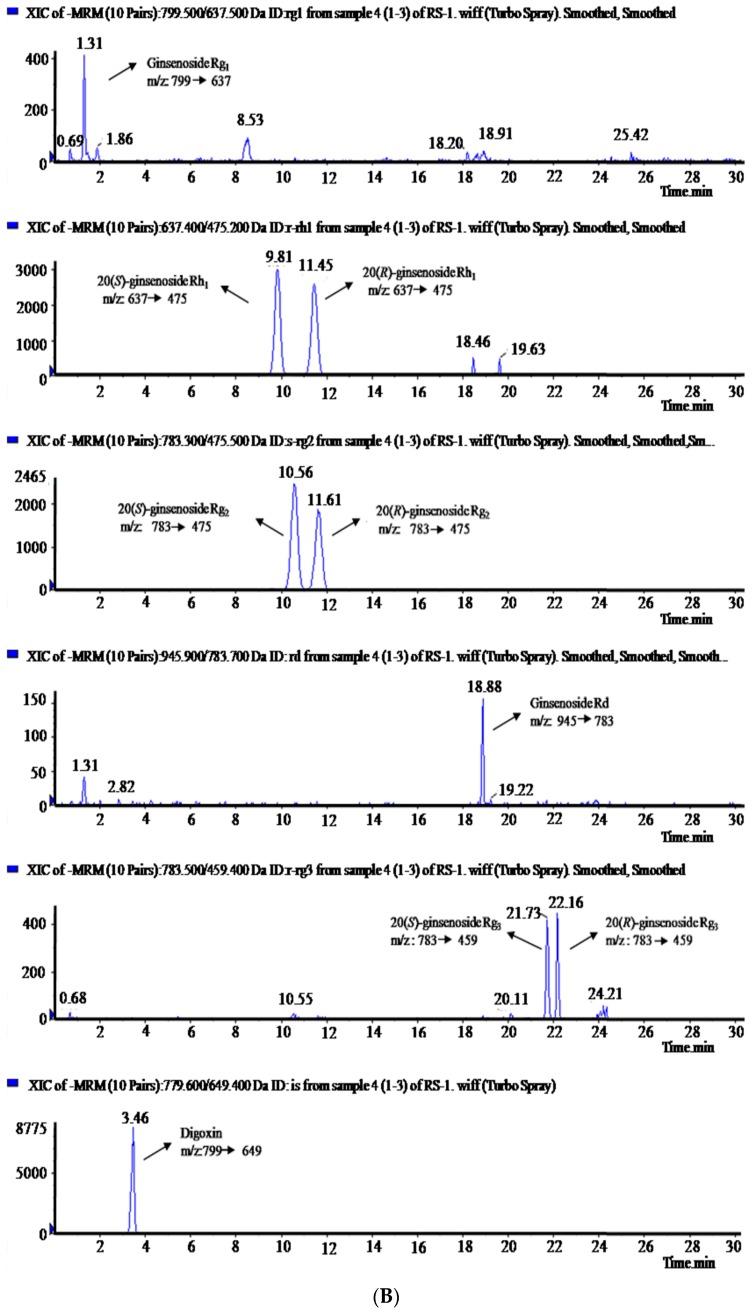
The extracted ionic currents: (**A**) blank plasma spiked with the eight ginsenoside standards and I.S. (digoxin); and (**B**) the plasma sample at 1 h after oral administration of 400 mg/kg GTSSL to rats spiked with I.S.

The extracted ionic currents (XIC) of the eight ginsenosides and digoxin as an internal standard (I.S.) on the reference standard, as well as in rat plasma, are shown in Figure 2A,B. For the XIC of blank rat plasma see Supplementary Materials, Figure S1. Mass spectral and tandem mass spectral measurements of the ginsenosides and I.S. were performed with these standard solutions by infusion. The eight standard ginsenosides were detected in both positive and negative ion modes. Since ginsenosides had not only higher sensitivity but also clearer mass spectra in the negative ion mode, data monitored in negative ion mode were used for the component detection and characterization, which made it easier to detect ginsenosides of lower content and confirm molecular ions or quasi-molecular ions in the identification of each peak. Deprotonated ions [M − H]^−^ at *m*/*z* 799 for **1**, 637 for **2** and **4**, 783 for **3** and **5**, 945 for **6**, 783 for **7** and **8**, and 779 for I.S., which showed up as base peaks in mass spectra, were selected as the precursor ions. The selection of product ions was accomplished by utilizing the “Quantitative Optimization” function of Analyst software. The product ions at *m/z* 637 [M − glucosyl (glu) − H]^−^ for **1**, 475 [M − glu − H]^−^ for **2** and **4**, 475 [M − rhamnosyl (rha) − glu − H]^−^ for **3** and **5**, 783 [M − glu − H]^−^ for **6**, 459 [M − 2glu − H]^−^ for **7** and **8**, and 649 [M − digitoxosyl − H]^−^ for I.S., were automatically selected at the highest peak intensity in tandem mass spectra.

### 2.2. Method Validation

#### 2.2.1. Selectivity and Linearity

From XICs of blank plasma spiked with analytes (A) and the plasma sample at 1 h after oral administration of 400 mg/kg GTSSL to rats spiked with I.S. (B) shown in Figure 2, and the XIC of blank plasma shown in Figure S1 (see Supplementary Materials section), it was indicated that there is no interfering peak in blank plasma under the assay conditions. The retention times (*R*_T_), the calibration curve, correlation coefficient (*r*^2^), linear range, lower limit of detection (LLOD), and lower limit of quantification (LLOQ) of each analyte were shown in Table 1. The *R*_T_ of I.S. was 3.46 min. Each analyte displayed good linearity.

**Table 1:** Calibration curves, LLODs, and LLOQs for eight ginsenosides.

Analyte	*R*_T_ (min)	Calibration Curve ^a^	Correlation Coefficient (*r*^2^)	Linear Range (ng/mL)	LLOD (ng/mL)	LLOQ (ng/mL)
**1**	1.24	*y* = 0.0101*x* + 0.0032	0.9935	0.45–126.60	0.12	0.45
**2**	8.51	*y* = 0.0122*x* + 0.0362	0.9909	0.30–85.54	0.07	0.30
**3**	9.12	*y* = 0.0028*x* + 0.0127	0.9943	0.45–129.60	0.12	0.45
**4**	9.98	*y* = 0.0105*x* + 0.0487	0.9959	0.21–59.62	0.05	0.21
**5**	10.02	*y* = 0.006*x* + 0.0409	0.9903	0.34–98.50	0.08	0.34
**6**	18.80	*y* = 0.0036*x* + 0.005	0.9921	0.95–182.40	0.27	0.95
**7**	21.29	*y* = 0.0008*x* + 0.0022	0.9980	0.32–93.31	0.08	0.32
**8**	21.72	*y* = 0.0009*x* + 0.0114	0.9910	0.38–108.86	0.09	0.38

**^a^** *y* = peak area and *x* = concentration (ng/mL).

#### 2.2.2. Precision, Accuracy and Stability

Intra- and inter-day precision and accuracy were determined by analyzing the extracted quality control (QC) standards at three concentration levels. Blank plasma were spiked with appropriate concentrations of ginsenosides, as well as 10 μL of I.S. solutions (5 µg/mL). The mixture was then processed as the drug-containing sample to prepare lower, middle, and upper QC samples.

The analysis was repeated on the same day and three consecutive days to give intra- and inter-day precision values that were expressed as relative standard deviation values (RSDs). Percentage differences were taken as measures of accuracy. The intra- and inter-day accuracies were within 81%–101% (see Supplementary Materials Table S1) and the precisions were within acceptable limits at three concentrations (*n* = 6). The stability of eight ginsenosides in rat plasma was tested as follows by assaying samples. The investigation of short-term stability was examined by analyzing the drug-containing samples at room temperature for 24 h and long-term stability was performed by analyzing the drug-containing samples stored at −20 °C for one month. Freeze/thaw stability was assessed in three cycles. The drug-containing samples were frozen at −20 °C for 24 h and thawed at 37 °C in water bath to be completely melted up to three cycles and then assayed. All stability QC samples were analyzed in six replicates. The stability in the test conditions were in the range of 80.01%–112.42% (see Supplementary Materials Table S2).

To determine extraction recovery and matrix effects, the extraction of rat plasma samples was also optimized in our preliminary studies by comparing protein precipitation reagents, such as methanol (MeOH), acetonitrile (MeCN), and normal butanol. The results were satisfactory when MeOH/MeCN = 4/1 (*v*/*v*) was used in protein precipitation. The mean extraction recoveries of eight ginsenoside in the test conditions were within 77%–96% (Table 2). The matrix effect is defined as the effect of co-eluting residual matrix components on the ionization of the target analyte. In other words, suppression or enhancement of analyte response is accompanied by diminished precision and accuracy of subsequent measurements. In this study, the matrix effects were in the range of 85.07%–93.90% (*n* = 6), which were within acceptable limits (Table 2) [29].

**Table 2:** Extraction recovery and matrix effects of eight ginsenosides.

Ginsenoside	Spiked (ng/mL)	Recovery		Matrix Effects	
		Measured (%)	RSD (%)	Measured (%)	RSD (%)
**1**	64.80	89.61	8.21	85.88	8.18
	16.20	88.52	12.86	86.98	7.67
	0.45	77.98	16.21	85.31	12.70
**2**	42.77	95.68	8.00	88.88	14.54
	10.69	90.94	12.27	85.28	14.24
	0.30	84.77	11.65	86.33	12.63
**3**	64.80	78.08	10.02	86.69	13.70
	16.20	83.79	12.51	87.06	14.16
	0.45	75.69	15.77	86.50	10.95
**4**	29.81	95.59	12.15	84.11	10.91
	7.45	83.75	13.63	85.07	14.23
	0.21	79.82	14.22	85.77	9.59
**5**	49.25	84.93	8.13	88.21	11.95
	12.31	87.84	10.95	85.79	11.83
	0.34	77.62	15.07	87.42	9.88
**6**	91.20	84.03	4.81	86.04	12.48
	22.80	83.20	11.74	85.64	13.25
	0.95	79.05	8.68	85.90	11.45
**7**	46.66	82.09	12.97	88.90	14.06
	11.66	79.91	11.51	93.90	8.68
	0.32	78.19	12.14	85.40	12.40
**8**	54.43	80.04	8.76	85.60	15.38
	13.61	77.51	11.96	87.22	11.44
	0.38	84.78	12.15	87.34	9.93

### 2.3. Pharmacokinetics of GTSSL

**Figure 3:**
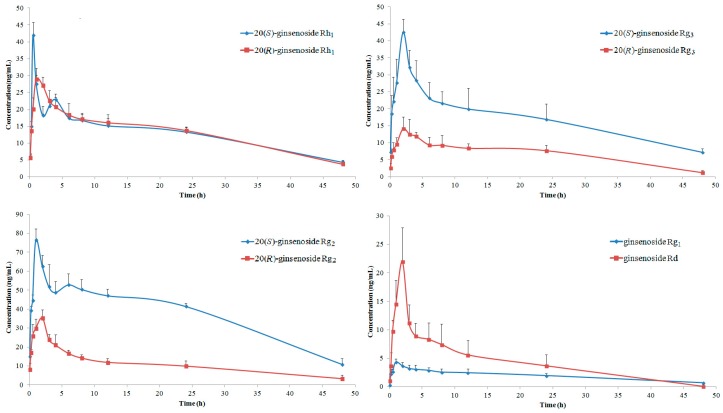
The concentration-time profiles of eight ginsenosides in GTSSL after oral administration at a dose of 400 mg/kg to rats (*n* = 6).

The oral median lethal dose (LD_50_) of GTSSL for mouse was estimated to be more than 5 g/kg, and GTSSL in a dose of 400 mg/kg produced a significant elongation of sleeping time of hexobarbital, as well as significantly inhibited writhing induced by 0.7% acetic acid. A dose-effect relationship has been established for the sedative, anti-inflammatory, and analgesic effects of GTSSL [30]. The mean plasma concentration-time profiles of ginsenosides **1**–**8** after oral administration of GTSSL powder at a dose of 400 mg/kg were shown in Figure 3. All eight ginsenosides were measurable in rat plasma up to 48 h after oral administration of GTSSL. Pharmacokinetic parameters were estimated using the DAS pharmacokinetic 2.0 software (Drug and Statistics 2.0, the Committee of the Mathematic Pharmacology, the Chinese Society of Pharmacology, Hefei, China). Maximum concentration (*C*_max_) and time to maximum concentration (*t*_max_) were the experimentally-observed values. Area under the plasma concentration–time curve (AUC) was calculated using the trapezoidal rule, and AUC_0__→t_ from time zero to real-time and AUC_0__→∞_ from time zero to infinity were calculated. Elimination half-life (*t*_1/2_) was calculated as *t*_1/2_ = 0.693/ke, and mean residence time (MRT) was calculated as AUMC/AUC. The estimated pharmacokinetic parameters of all analytes after oral administration were shown in Table 3.

The ginsenosides **6**, **7**, and **8**, belonging to protopanaxadiol-type saponins, exhibited relatively long *t*_max_ values, suggesting that they were slowly absorbed. The ginsenosides **1**–**5**, belonging to protopanaxatriol-type saponins, had different *t*_max_ values, which should be due to the differences in the substituted groups. According to the PK investigations on 20(*R*)-ginsenoside Rg_3_ (**8**) following oral administration of the single **8** to rats [31], the value of *t*_max_ was (4.40 ± 1.67) h, suggesting promotion of absorption by oral administration of GTSSL. Namely, the *t*_max_ of **8** after oral administration of GTSSL is shorter than that of single **8** administration, the oral administration of GTSSL containing **8** resulted in a shorter *t*_max_ of **8** than when the single **8** was administered. It has also been noted that **3** (20(*S*)-ginsenoside Rg_2_) was not detected in the rat plasma samples after oral administration of single **3** with a dose at 10 mg/kg [32], conflicting with the results obtained in this research and further research is needed. Taking into consideration combined with results from our present research, it was believed that absorption of the combination as found in herbal preparations would be superior to that of individual compound *in vivo*, which the increased absorption of **3** might be due in part to the additional compound absorption interaction and GTSSL could significantly enhance the exposure of **3** in rat plasma.

**Table 3:** The pharmacokinetic parameters of eight ginsenosides in GTSSL after oral administration at a dose of 400 mg/kg to rats (*n* = 6).

Ginsenoside	AUC_0→_*_t_* (μg h/L)	AUC_0→∞_ (μg h/L)	MRT_0→*t*_ (h)	MRT_0→∞_ (h)	*t*_1/2_ (h)	*t*_max_ (h)	C_max_ (μg/L)
**1**	88.77 ± 22.08	105.64 ± 18.32	9.78 ± 0.48	12.70 ± 2.98	14.99 ± 3.77	2	4.32 ± 1.09
**2**	622.92 ± 46.37	753.19 ± 48.85	17.06 ± 0.14	27.61 ± 1.67	20.61 ± 1.18	0.5	41.99 ± 3.82
**3**	1778.47 ± 86.07	2302.22 ± 536.47	17.43 ± 0.55	25.65 ± 4.02	22.24 ± 9.88	1	76.42 ± 5.90
**4**	616.55 ± 49.28	716.65 ± 92.94	17.05 ± 0.67	24.78 ± 3.58	17.68 ± 2.99	1	29.74 ± 2.71
**5**	511.51 ± 63.84	617.80 ± 156.78	15.63 ± 1.92	25.55 ± 10.45	19.05 ± 8.05	2	35.68 ± 4.04
**6**	218.10 ± 18.69	221.75 ± 16.57	12.43 ± 1.46	13.46 ± 2.11	7.30 ± 3.32	2	22.05 ± 2.21
**7**	778.63 ± 56.72	922.70 ± 119.85	16.50 ± 1.02	23.83 ± 2.09	19.11 ± 3.34	2	42.91 ± 3.55
**8**	302.59 ± 32.45	311.84 ± 30.37	15.43 ± 0.74	16.91 ± 1.39	9.93 ± 2.34	2	15.42 ± 3.38

Compounds **2** and **4**, **3** and **5**, **7** and **8** are three pairs of *R*/*S* epimerics at C-20 (Figure 1). It was interesting that the *t*_1/2_ of 20(*S*)-epimers were always longer than those of 20(*R*)-epimers and showed significant differences, indicating that elimination of 20(*S*)-epimer from rat body was more slower than that of the 20(*R*)-epimer and 20(*S*)-epimer would be expected to have a longer-lasting effect. Although the MRT_0__→*t*_ values of three pairs of *R*/*S* epimerics were comparable, the MRT_0→∞_ of **7** showed nearly 1.5 times larger than that of **8**. The *t*_1/2_ values of **6**, **8**, and **1** were (7.30 ± 3.32), (9.93 ± 2.34) and (14.99 ± 3.77) h, respectively, indicating that they were metabolized slowly but still more rapidly than **2**–**5** and **7**. These results were consistent with their MRT_0→∞_ profiles. MRT represents the average time a drug molecule remains in the body after administration. It has been well known that the larger MRT could provide a longer duration of action for drugs. However, whether these superior benefit will actually translate to humans requires further study.

Compared to the previous PK studies of individual ginsenoside Rd [33,34], ginsenoside Rg_1_ [35], 20(*R*)- and 20(*S*)-ginsenoside Rg_2_ [8], and 20(*R*)-ginsenoside Rg_3_ [31,36,37] after intravenous administration to animals or healthy volunteers, we have investigated three couples of 20(*R*)- and 20(*S*)-epimers simultaneously by LC-ESI-MS/MS, which possesses higher sensitivity and precision. On the contrary, the PK studies of individual ginsenoside was simple and uncomplicated, it is well-known that interactions from complex constituents of traditional Chinese medicines could substantially affect the PK profiles of targeted compounds, therefore, the research of PK on eight ginsenosides of GTSSL here will be useful for drug development in future.

## 3. Experimental Section

### 3.1. Materials and Reagents

GTSSL was prepared by using standard method as previously published [38]. The eight reference standard ginsenosides, ginsenoside Rg_1_ (**1**), 20(*S*)-ginsenoside Rh_1_ (**2**), 20(*S*)-ginsenoside Rg_2_ (**3**), 20(*R*)-ginsenoside Rh_1_ (**4**), 20(*R*)-ginsenoside Rg_2_ (**5**), ginsenoside Rd (**6**), 20(*S*)-ginsenoside Rg_3_ (**7**), and 20(*R*)-ginsenoside Rg_3_ (**8**) were purified from acid hydrolysates of GTSSL [5,6,39]. Their chemical structures as shown in Figure 1 were confirmed by MS, ^1^H- and ^13^C-NMR spectroscopies and the purities were all >99.8% analyzed with HPLC-DAD method. The I.S. with a purity of 99.9% was purchased from the National Institutes for Food and Drug Control (Beijing, China). LC-MS grade MeCN and MeOH was obtained from J. T. Baker (Center Valley, PA, USA). HPLC-grade formic acid was purchased from Dikma Tech. Inc. (Beijing, China). Water (H_2_O) was purified by a Milli-Q system (Millipore, Billerica, MA, USA) in our laboratory. Heparin sodium injection (Lot# 20110101) was purchased from Tianjin Biochemical Pharmaceutical Co., Ltd. (Tianjin, China) and normal saline was from Shuanghe Pharmaceutical Co. (Beijing, China). Other reagents were of analytical grade.

### 3.2. Animals

Male Sprague–Dawley (SD) rats weighting 200–220 g were supplied by the Laboratory Animal Center of Peking University Health Science Center (Beijing, China). The rats were kept in a controlled breeding room with temperature conditions at (22 ± 1) °C and relative humidity at (60 ± 5)% before the experiment. They were fed standard laboratory chow with water *ad libitum* for three days and then fasted with free access to water for 12 h prior to each experiment. All experimental procedures were approved by the Animal Care Ethics committee on Peking University (No. LA2014162) and conducted according to the European Community guidelines for the use of experimental animals.

### 3.3. Instrumental and Chromatographic Conditions

Detection and quantification of the analytes were performed on a HPLC–MS system. The analytical DIONEX Ultimate 3000 HPLC system was equipped with an Ultimate 3000 Pump, a DIONEX Ultimate 3000 Autosampler and a DIONEX Ultimate 3000 Compartment. The chromatograph was connected online to a 4000QTRAP triple quadrupole tandem mass spectrometer (Applied Biosystems/MDS Sciex, Toronto, ON, Canada) equipped with an electrospray ionization (ESI) source for the mass analysis and detection. Analyst 1.5.1 software (Applied Biosystems/MDS Sciex) was used for data collection and analysis. The separation was performed on a BDS HYPERSIL C_18_ column (100 × 2.1 mm i.d., 2.4 μm; Thermo Scientific, Massachusetts, MA, USA). The mobile phases were MeCN (A) and H_2_O (B) with gradient condition of A as follows: 0–16 min, 25%; 16–16.5 min, 25%–42%; 16.5–19.5 min, 42%; 19.5–22 min, 42%–50%; 22–22.1 min, 50%–90%; 22.1–28 min, 90%; 28–28.1 min, 90%–25%; 28.1–34 min, 25%. The flow rate was set at 0.4 mL/min and the injection volume was 5 µL. Quantification of the analytes was performed using multiple reaction monitoring (MRM) in the negative ionization mode with the following settings after optimization: source voltage 3.5 kV, nebulizer gas 50 psi, turbo gas 70 psi, curtain gas 35 psi, collision gas 7, and source temperature (TEM) 500 °C. Gases were 99.999% nitrogen. The MRM transitions monitored were from *m*/*z* 799.5 to 637.5 for **1**, from *m*/*z* 637.5 to 475.1 for **2**, from *m*/*z* 783.3 to 475.5 for **3**, from *m*/*z* 637.6 to 475.2 for **4**, from *m*/*z* 783.4 to 475.6 for **5**, from *m*/*z* 945.9 to 783.7 for **6**, from *m*/*z* 783.6 to 459.5 for **7**, from *m*/*z* 783.7 to 459.6 for **8**, and from *m*/*z* 779.6 to 649.4 for I.S. Data acquiring and processing were performed with Analyst software, version 1.6 (Applied Biosystems/MDS Sciex).

### 3.4. Identification and Determination of Ginsenosides in Blood Samples

#### 3.4.1. Preparation of Blood Samples

The rat blood samples were processed to obtain plasma by centrifugation at 3000 *g* for 10 min. A 150 μL aliquot of the upper layer of plasma was mixed with 10 μL of I.S. (5 μg/mL) and 750 μL MeOH/MeCN = 4/1 (*v*/*v*) using vortex-mixing for 1 min and then centrifuged at 10,000 *g* for 10 min at 4 °C. The upper layer was transferred to another clean Eppendorf tube and dried under gentle N_2_ gas stream at 45 °C. Each dried residue sample was added to 120 μL of MeOH, using vortex-mixing for 1 min and then centrifuged at 10,000 *g* for 10 min at 4 °C. A 5 μL aliquot of supernatant was injected into the LC-ESI-MS/MS system for analysis. The same sample handling process was used for the recovery and precision determinations.

#### 3.4.2. Preparation of Stock Solutions, Calibration Samples and Quality Control Samples

Stock solutions of the eight reference standard ginsenosides were separately prepared in MeOH. A 5 μg/mL solution for I.S. was also prepared in MeOH. Eight calibration curves for ginsenosides were prepared with the method above by spiking blank rat plasma with I.S. A mixed working solution containing eight analytes at seven concentrations, which were obtained by dilution the stock solutions, to give 126.60, 64.80, 32.40, 16.20, 5.40, 1.35, and 0.45 ng/mL for **1**; 85.54, 42.77, 21.38, 10.69, 3.56, 0.89, and 0.30 ng/mL for **2**; 129.60, 64.80, 32.40, 16.20, 5.40, 1.35, and 0.45 ng/mL for **3**; 59.62, 29.81, 14.90, 7.45, 2.48, 0.62, and 0.21 ng/mL for **4**; 98.50, 49.25, 24.62, 12.31, 4.10, 1.03, and 0.34 ng/mL for **5**; 182.40, 91.20, 45.60, 22.80, 11.40, 2.85, and 0.95 ng/mL for **6**; 93.31, 46.66, 23.33, 11.66, 3.89, 0.97, and 0.32 ng/mL for **7**; and 108.86, 54.43, 27.22, 13.61, 4.54, 1.13, and 0.38 ng/mL for **8**. QC samples at lower, middle and upper concentrations were chosen as the seventh, fourth, and second concentration solution. All the solutions were stored at −20 °C before use.

### 3.5. Method Validation

#### 3.5.1. Linearity and Selectivity

Each standard curve consisted of seven concentration levels mentioned above and was constructed by calculating the peak area ratio (*y*) of an analyte to I.S. against the analyte concentrations (*x*). The correlation coefficient (*r*^2^) of the calibration curves should be >0.990 to satisfy linearity requirements. The LLOD was defined as the amount that could be detected with a *S*/*N* of three. The LLOQ for analyte of the assay was defined as the lowest concentrations of the calibration curve that could be quantitated with a *S*/*N* of at least 10. Each concentration standard needed to meet the following acceptable criteria: accuracy should not exceed 20%. The selectivity of the method was investigated by analyzing blank rat plasma, blank plasma spiked with each standard analyte and I.S., and a rat plasma sample.

#### 3.5.2. Precision and Accuracy

The precision and accuracy were analyzed with the QC samples (lower, middle, and upper concentration) in six replicates, which were assessed on three continuous days. The intra- and inter-day precision was evaluated from the relative standard deviation (RSD %) and the relative error (RE%) was calculated according to the formula:

RE% = [(assayed value − nominal value)/nominal value] × 100%(1)

#### 3.5.3. Extraction Recovery and Matrix Effects

The extraction recoveries of analytes were assessed by comparing the mean peak areas of QC samples (dissolved in MeOH) with the mean peak areas of the spike-after-extraction samples (blank plasma extracted with MeOH/MeCN = 4/1) at the same concentrations (*n* = 6). The matrix effects of analytes were assessed with comparing the mean peak areas of QC samples (dissolved in MeOH) with the mean peak areas of the analytes in QC samples which were dissolved in the blank plasma extracted with MeOH/MeCN (4/1) at the same concentrations (*n* = 6). There was no matrix effect if the ratio was between 85% and 115%, however the matrix effect was obvious if the ratio was less than 85% or more than 115% [29].

#### 3.5.4. Stability

The stability was investigated by assessing six replicates of the QC samples in different conditions. These conditions included storage 24 h at indoor temperature, three freeze-thaw cycles, and storage in the dark for 30 days at −20 °C. The samples were considered stable if the RE% was within 15% of the actual value.

### 3.6. Application

Male SD rats were used in the study after a one week acclimatization period. The rats were allowed free access to food and water before the experiment, and then fasted with free access to water for 12 h prior to each experiment. After overnight fasting, GTSSL powder was administered orally at the dosage of 400 mg/kg to rats (*n* = 6). The blood samples (0.5 mL) were collected in heparinized tubes at 0.083, 0.25, 0.5, 1, 2, 3, 4, 6, 8, 12, 24, and 48 h after dosing. The blood samples were immediately centrifuged at 3000 *g* for 10 min, and the supernatant plasma layer was gathered and stored at −20 °C until analysis.

## 4. Conclusions

The LC-ESI-MS/MS method was developed for identification and quantification of eight ginsenosides of GTSSL: ginsenoside Rg_1_ (**1**), 20(*S*)-ginsenoside Rh_1_ (**2**), 20(*S*)-ginsenoside Rg_2_ (**3**), 20(*R*)-ginsenoside Rh_1_ (**4**), 20(*R*)-ginsenoside Rg_2_ (**5**), ginsenoside Rd (**6**), 20(*S*)-ginsenoside Rg_3_ (**7**), and 20(*R*)-ginsenoside Rg_3_ (**8**), in rat plasma with high sensitivity, accuracy, precision, selectivity, and rapidity. The PK profiles of these eight ginsenosides were first simultaneously determined following oral administration of a single dose to rats. The PK information on these ginsenosides obtained in this study could provide valuable reference for the clinical use of GTSSL.

## Supplementary Materials

The XIC of blank rat plasma, as well as intra- and inter-day accuracy, precision and stability of method validation for the analysis of eight ginsenosides in rat plasma are available as Supplementary Materials, which may be accessed at: http://www.mdpi.com/1420-3049/20/12/19790/s1.
