# Supplementary material for: Simultaneous Determination of Eight Ginsenosides in Rat Plasma by Liquid Chromatography–Electrospray Ionization Tandem Mass Spectrometry: Application to Their Pharmacokinetics
Source: Molecules. 2015 Dec 3;20(12):21597–608. doi: 10.3390/molecules201219790 (PMC6332349; doi:10.3390/molecules201219790)
Supplement: Supplementary file 1 [file molecules-20-19790-s001.pdf]

Supplementary Materials: Simultaneous Determination of 8 Ginsenosides in Rat Plasma by Liquid Chromatography–Electrospray Ionization Tandem Mass Spectrometry: Application to Their Pharmacokinetics

Li-Yuan Ma, You-Bo Zhang, Qi-Le Zhou, Yan-Fang Yang and Xiu-Wei Yang

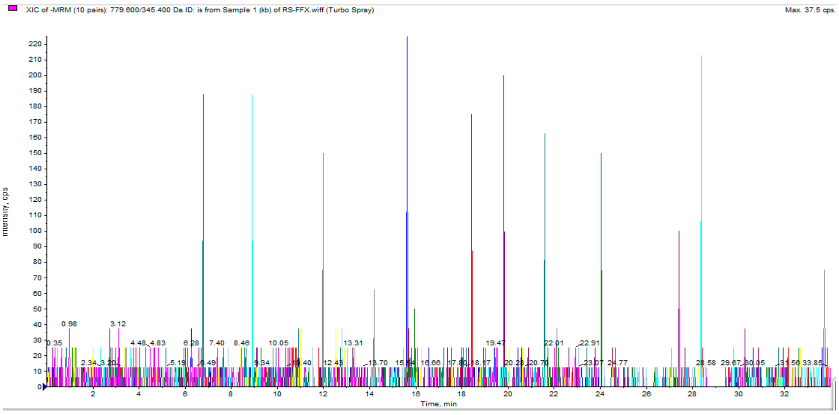

Figure S1. The XIC of blank rat plasma.

Table S1. Intra- and inter-day accuracy and precision of 8 ginsenosides in rat plasma (n = 6).

Ginsenoside	Spiked (ng/mL)	Intra-Day			Inter-Day		
		Measured (ng/mL)	RSD (%)	Accuracy (%)	Measured (ng/mL)	RSD (%)	Accuracy (%)
	64.80	59.22	5.89	91.39	56.40	6.23	87.04
**1**	16.20	14.45	3.43	89.22	14.14	12.98	87.30
	0.45	0.40	10.93	89.31	0.40	9.82	88.15
	42.77	39.96	3.88	93.44	37.84	6.20	88.48
**2**	10.69	10.44	14.18	97.62	9.41	14.87	88.02
	0.30	0.28	15.07	94.96	0.27	7.03	90.79
	64.80	60.25	8.16	92.98	58.92	6.37	90.92
**3**	16.20	14.06	2.14	86.80	14.85	14.90	91.65
	0.45	0.41	13.98	91.20	0.42	10.37	93.78
	29.81	26.90	6.25	90.24	25.64	11.89	86.01
**4**	7.45	6.98	8.39	93.75	6.94	5.42	93.12
	0.21	0.18	8.71	87.89	0.17	13.92	81.16
	49.25	45.81	4.72	93.01	45.81	4.67	93.99
**5**	12.31	10.97	5.58	89.09	10.57	6.62	86.49
	0.34	0.30	12.33	88.30	0.30	9.63	88.64
	91.20	86.06	4.22	94.37	87.46	5.78	95.89
**6**	22.80	19.40	13.27	85.09	19.51	10.74	85.57
	0.95	0.82	7.00	85.91	0.79	8.07	83.15
	46.66	40.45	4.78	86.69	41.35	6.15	88.61
**7**	11.66	10.40	11.37	100.89	11.47	13.71	98.36
	0.32	0.33	11.37	100.89	0.31	18.40	96.66
	54.43	51.54	4.07	94.69	48.68	7.60	89.44
**8**	13.61	11.87	11.07	87.21	11.60	7.33	85.20
	0.38	0.32	15.79	83.09	0.33	16.14	87.11

Table S2. Stability of 8 ginsenosides in rat plasma.

Ginsenoside	Spiked (ng/mL)	Short-Term Stability			Freeze/Thaw Stability			Long-Term Stability		
		Measured (ng/mL)	RSD (%)	Accuracy (%)	Measured (ng/mL)	RSD (%)	Accuracy (%)	Measured (ng/mL)	RSD (%)	Accuracy (%)
**1**	64.80	52.14	5.56	80.47	56.14	6.07	86.63	54.88	13.42	84.69
	16.20	14.75	14.50	91.03	12.96	8.89	80.01	13.59	14.77	83.87
	0.45	0.36	19.63	80.55	0.38	12.33	85.21	0.36	15.24	80.28
**2**	42.77	34.29	14.29	80.16	34.48	7.87	80.61	42.09	2.92	98.42
	10.69	11.15	14.72	104.33	9.08	14.99	84.90	9.58	8.52	89.61
	0.30	0.26	19.51	86.25	0.25	15.39	84.50	0.27	13.41	90.23
**3**	64.80	60.33	8.86	93.10	60.97	14.09	94.09	57.70	4.17	89.05
	16.20	15.41	11.25	95.13	15.97	13.48	98.57	14.91	7.63	92.03
	0.45	0.38	19.55	83.72	0.37	14.43	81.81	0.41	16.33	91.00
**4**	29.81	30.13	8.42	101.07	27.23	14.66	91.33	25.58	14.90	85.80
	7.45	6.36	10.83	85.37	6.57	12.07	88.14	6.02	13.48	80.87
	0.21	0.20	19.49	95.07	0.17	19.46	84.25	0.19	10.22	90.71
**5**	49.25	45.91	4.72	93.21	44.19	5.96	89.72	43.65	9.29	88.62
	12.31	12.92	13.72	104.96	12.80	12.45	104.02	10.49	4.92	85.19
	0.34	0.30	19.71	89.46	0.37	19.10	109.56	0.29	14.14	85.92
**6**	91.20	96.54	7.03	105.86	89.60	8.74	98.25	81.44	13.74	89.30
	22.80	19.89	13.16	87.26	18.53	10.40	81.28	18.79	14.49	82.43
	0.95	0.79	16.57	83.36	0.77	17.71	80.93	0.78	16.33	82.48
**7**	46.66	38.57	12.12	82.66	39.99	8.58	85.69	39.73	15.52	85.14
	11.66	11.99	14.88	102.84	10.49	13.97	90.01	11.24	11.12	96.41
	0.32	0.36	18.02	112.42	0.35	19.41	106.96	0.32	18.24	99.32
**8**	54.43	46.86	11.03	86.09	46.04	3.89	84.59	45.96	13.62	84.44
	13.61	11.13	8.72	81.78	10.91	10.60	80.13	12.07	6.73	88.65
	0.38	0.41	12.30	108.31	0.31	19.86	81.23	0.36	7.10	94.32
